# Machine Learning-Based Insights into Environmental Determinants of *Morchella importuna* Growth in Muğla, Türkiye

**DOI:** 10.3390/life15121806

**Published:** 2025-11-25

**Authors:** Hakan Allı, Nevin Güler Dincer, Aytaç Pekmezci

**Affiliations:** 1 Department of Biology, Faculty of Science, Mugla Sıtkı Kocman University, Muğla 48000, Türkiye; ahakan@mu.edu.tr; 2 Department of Statistics, Faculty of Science, Mugla Sıtkı Kocman University, Muğla 48000, Türkiye; nguler@mu.edu.tr

**Keywords:** morel, *Morchella importuna*, machine learning algorithms, feature importance

## Abstract

*Morchella* species are edible mushrooms with high nutritional, ecological, and economic significance. Due to overharvesting and habitat loss, their natural populations face serious threats, which has led to growing interest in controlled cultivation. Although several studies have investigated *Morchella* growth, particularly *M. importuna*, key uncertainties remain regarding the environmental and nutritional requirements for optimal cultivation. Previous studies have largely focused on experimental approaches, but systematic machine learning analyses remain scarce. This study aims to fill this gap by applying machine learning algorithms to identify the most critical soil and climatic variables affecting *M. importuna* growth. To achieve this objective, using soil and climatic data from three localities in Türkiye, 20 features and *M. importuna* growth were analyzed by following a three-step procedure: (i) applying regression analysis based on eleven methods, (ii) selecting best fit model by using three goodness of fit measures, and (iii) calculating feature importances based on permutation importance and Shapley additive explanations methods. Gradient Boosting emerged as the best-performing algorithm, highlighting lime (%), phosphorus, precipitation, magnesium, minimum temperature, and maximum temperature as the most important predictors of growth. These results provide quantitative evidence that can guide cultivation strategies for morels, contributing to both conservation and commercial production.

## 1. Introduction

*Morchella* Fr. species (morels), are a type of edible mushroom that is highly valued for its distinctive flavor and appearance. Morels have a unique appearance with a honeycomb-like cap, which is usually elongated and dimpled. The color can range from light yellow to dark brown [1,2]. *Morchella* species typically occur in forested areas, especially in spring, usually between March and May in the Northern Hemisphere (except *Morchella galilaea* Masaphy & Clowez). The only species that grows in autumn is *Morchella galilaea* Masaphy & Clowez. The vast majority of morels are mycorrhizal fungi, meaning that they form a symbiotic relationship with the roots of certain trees [3]. Growing under certain pine trees (e.g., *Morchella esculenta* (L.) Pers., *M. elata* Fr.), morels prefer well-drained soil and some shade. They like moist soils with a pH range of 6.0 to 7.5 and not wet. However, morel mushrooms also thrive in many different areas, such as construction waste (*M. importuna* M. Kuo, O’Donnell & T.J. Volk), fire areas (e.g., *M. eximia* Boud., *M. exuberans* Clowez, Hugh Sm. & S. Sm.), wood stores (*M. importuna*), in wood shavings (*M. rufobrunnea* Guzmán & F. Tapia), and gypsum soil (*M. steppicola* Zerova), etc [4,5,6,7,8].

When growing morel mushrooms, it is essential to mimic their natural habitat as much as possible. This involves using the right type of soil that ensures proper drainage and nutrient availability. In addition, maintaining optimum soil moisture is crucial for successful cultivation. Some common soil types used for morel mushroom cultivation include sandy loam, loamy soil, and soil mixed with organic materials such as compost or peat moss. These soil types provide the necessary structure, nutrients, and moisture retention needed for morel mushrooms to thrive. In summary, cultivating morel mushrooms requires specific attention to soil features such as drainage, pH, organic matter content, and moisture levels to create a suitable environment for their growth [9]. Furthermore, research indicates that soil microorganisms play crucial roles in supporting plant growth morel. Stimulation of ascocarp formation during growth is very effective for the growth of morel [10]. In contrast, some bacteria and microfungi in soil can act as pathogens of fungi, leading to reduced yields and serious quality loss [11]. Studies on the culture of *Morchella* have been ongoing for more than 100 years, and the first production was realized in China in 2012. However, since then, some uncertainties (such as oxidative stress, sclerotia formation, and pigmentation) have prevented morel cultivation, and studies on its culture are still ongoing today [12,13,14,15]. The soil microbiome at all stages of morel mushroom growth is very important, as various biomarkers can be produced and ecologically produced [16]. In addition, nutrition, humidity, temperature, shade, and light play an important role in the ascocarp formation of morel mushroom [13]. All these make it important in determining the growing environment and requirements of morel mushroom [14].

*Morchella* species, which grows naturally in many regions of Türkiye as well as in the world, grows abundantly, especially in the Mediterranean climate zone, where our country is located, and is collected and exported to the world from here. *Morchella* species have the potential to trigger technological and organizational innovations in areas such as their high economic value [17], long-term storage possibilities, and their use in the food sector and tourism sectors. Turkey generates approximately $2 million in annual revenue from morels exports [17]. Considering that the awareness of proper and balanced nutrition is becoming more and more important day by day, *Morchella* species, which are the source of various phytochemicals such as phenolic compounds, tocopherols, ascorbic acid, and vitamin D [18], which are critical factors in proper nutrition, are collected from natural coniferous forests by local people and consumed for food purposes. However, there are some problems in the studies on the cultivation of morel mushroom, especially the *M. importuna* species, which is consumed in 28 countries and of which India, Pakistan, Türkiye, Nepal, Bhutan, USA, Canada, and China are the biggest exporters [14]. However, the biggest problem is the continuing significant uncertainty regarding the environmental and nutritional requirements necessary for optimal cultivation.

This study aimed to determine the fundamental environmental and soil factors affecting the prediction of *M. importuna* growth by applying machine learning algorithms. In fact, the main hypothesis of this study is whether 20 features reflecting the characteristics of the soil and environmental conditions of the sampled under study have an effect on the prediction of *M.importuna* growth. For this purpose, the following hypotheses are investigated for each feature used in the study by using permutation importance and Shapley additive explanation (SHAP) methods.

**Hypothesis** **1.** *Feature A is not an effective feature for predicting growth of M. importuna.*

**Hypothesis** **2.** *Feature A is an effective feature for predicting growth of M. importuna*.

It is expected that results obtained from the hypotheses given will contribute to both conservation and commercial production by providing quantitative evidence that can guide cultivation strategies for mushrooms.

## 2. Materials and Methods

### 2.1. Material and Study Area

One of the most widely used morel species, *M. importuna*, is the subject of this investigation. Among morels, *M. importuna* is an edible species. It is currently growing [19], and despite its high cost, its demand in the global commercial sector is steadily rising [20]. It has been observed that *M. importuna* grows natively in western North America, Spain, France, Israel, China, India, Pakistan, Türkiye, and the temperate parts of the Northern Hemisphere, which are its most abundant natural habitats [20,21,22]. Because of its potential in comparison to other morel species, the data collected indicate that *M. importuna* is the most desired species in controlled morel cultivation worldwide [14,19]. Common locations for this species include sawdust beds, gardens, the perimeter of building sites, and other urban areas [23]. Approximately 80–90% more *M. importuna* is cultivated in China than other species. Fresh morel yields of 0–7620 kg per hectare have been reported in the majority of China’s provinces where cultivation is common [9].

**Figure 1:**
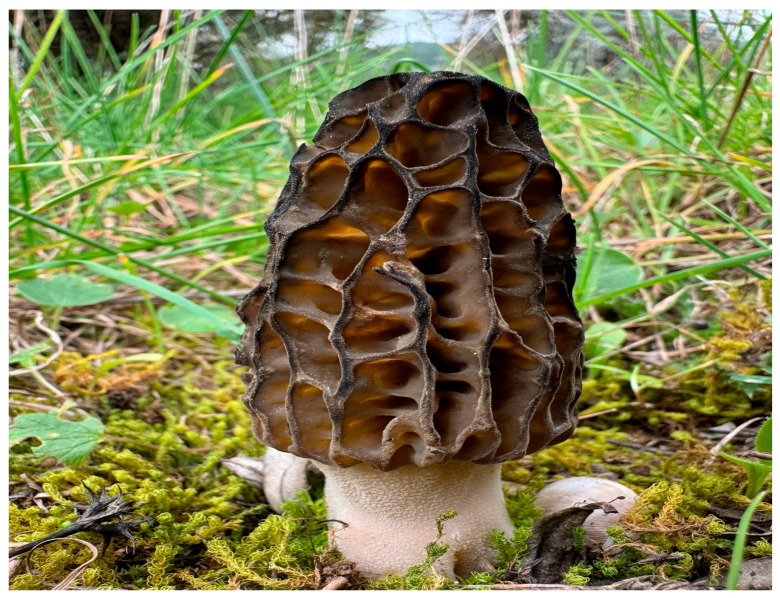
*Morchella importuna* (Photograph: by H. ALLI).

One of the few species of morel that has been effective in culture studies and grows in a variety of environments is *M. importuna*, which is a member of the class Pezizomycetes, order Pezizales, and family Morchellaceae (Figure 1).

**Figure 2:**
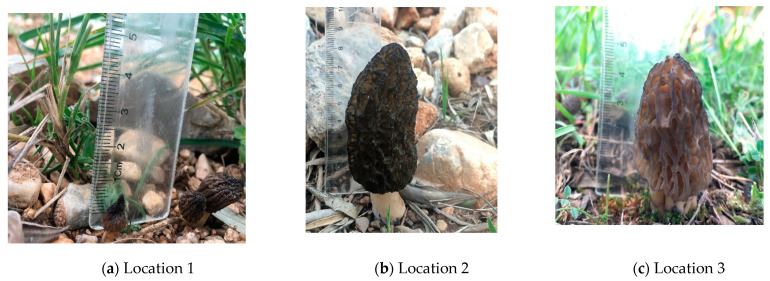
*M. importuna* growth stages in 3 different environments (Photograph: by H. ALLI).

In this study, morel samples were collected from 3 different regions close to each other in Muğla-Ortaköy. These localities are Locality 1: 370 10′ 42″ N, 280 25′ 58″ E, 637 m; Locality 2: 370 10′ 42″ N, 280 26′ 3″ E, 632 m; Locality 3: 370 10′ 43″ N, 280 26′ 5″ E, 637 m. Soil samples are taken at a depth of 20 cm from the localities where morels are collected and analyzed in the accredited Soil, Plant, and Water Analysis Laboratory of Muğla Sıtkı Koçman University (MSKU) Research Laboratories Center. In addition, the length of the samples (cm) is measured and photographed with a ruler at the same time every day for 15 days (Figure 2). 

**Figure 3:**
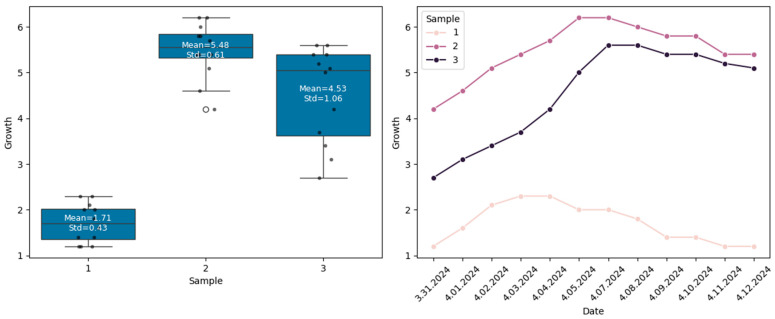
The *M. importuna* growth according to samples.

Figure 3 shows the box-plot and line graphs for the *M. importuna* growth (length) according to samples.

According to Figure 3, the lowest and highest growth rates are observed in samples 1 and 2, respectively. When examining the change in *M. importuna* growth against time, it is observed that the length of *M. importuna* increases for the first 4 days in sample 1, for the first 6 days in sample 2, and for the first 7 days in sample 3, and then decreases.

### 2.2. Machine Learning Algorithms

This study utilizes regression analysis within machine learning algorithms to predict *M. importuna* growth. A dependent variable and k features (independent variables) may be discrete, continuous, or categorical. When the dependent variable is continuous, regression analysis is used to estimate the relationship between the dependent variable and the features (independent variables). The estimated model predicts the dependent variable (*M. importuna* growth in this study) based on the values of the k features given.

No single regression analysis method is perfect for all data sets. To identify the most suitable method for the data set, various methods are applied, and the optimal one is selected based on different goodness of fit (GoF) measures. In this study, eleven regression analysis methods, including AdaBoost (AB) [24], Decision Tree (DT) [24,25,26], XGBoost (XGB) [27], Extra Trees (ET) [25], Random Forest (RF) [25,27,28], Gradient Boosting (GB) [25,28], K-Nearest Neighbors (KNN) [26,28], HistGradient Boosting (HGB) [29], Ridge Regression (RR), Lasso Regression (LR) [30], and Linear Regression(LiR), are applied to the data set, and GoF measures given in Section 2.3 are used to determine the model that provided the best fit in predicting *M. importuna* growth.

### 2.3. Goodness of Fit Measures

In this study, Mean Absolute Error (MAE), Mean Absolute Percentage Error (MAPE), and R^2^ are selected as GoF measures. The measures are defined as follows:

(1) $$R^{2}=1-\frac{{\sum_{i=1}^{n}\left(Y_{actual,i}-Y_{predicted,i}\right)}^{2}}{{\sum_{i=1}^{n}\left(Y_{actual,i}-{\bar{Y}}_{actual}\right)}^{2}}$$

(2) $$MAE=\frac{\sum_{i=1}^{n}\left|Y_{actual,i}-Y_{predicted,i}\right|}{n}$$

(3) $$MAPE=\frac{1}{n}(\sum_{i=1}^{n}\left|\frac{Y_{actual,i}-Y_{predicted,i}}{Y_{actual,i}}\right|)$$

where $Y_{actual,i}$ is ith actual value of *M. importuna* growth, ${\bar{Y}}_{actual}$ is mean of actual values, $Y_{predicted,i}$ is ith predicted value obtained from the models, and n is the number of observations. The model provided the smallest MAE and MAPE values, and the highest $R^{2}$ value is selected as the best fit model.

### 2.4. Feature Importance

This study uses permutation importance and SHAP methods [31] to measure the contribution of each feature to the predicted model. To calculate the permutation importance score of feature j, firstly, a reference score (RS) is calculated by fitting a predictive model to the original data set. At the next step, the values of feature j to calculate its importance are randomly shuffled, while the values of other features values remain stable. A predictive model is fitted to the new data set and ${RS}_{i,j}\;$(i = 1, 2, …, k) is calculated again. This process is repeated k times for feature j and the importance is calculated as follows [32]:

(4) $$I_{j}=RS-\;\frac{1}{k}\sum_{i=1}^{k}{RS}_{i,j}$$

Positive feature importance ratings showed that the feature reduced prediction errors, but negative feature importance scores showed that the feature increased prediction errors [33].

It is used in SHAP to measure the impact of a feature on the predictions [31,34,35]. SHAP was proposed by Lundberg and Lee [31], inspired by game theory. Features correspond to players while predictions correspond to payouts. SHAP measures the contribution of each player, i.e., feature, to the game, i.e., predictions. SHAP values are calculated as follows:

(5) $$\emptyset_{i}=\sum_{K\subseteq M\{i\}}\frac{\left|K\right|!\left(N-\left|K\right|-1\right)!}{N!}\left[g_{x}\left(K\cup \left\{i\right\}\right)-g_{x}(K)\right]$$

(6) $$g_{x}\left(K\right)=E[\left.g(x)\right|x_{K}]$$

Where K is a subset of the features, M is the set of all features, and $g_{x}\left(K\right)$ is expected value of the g(x) on the subset K [34]. Here, negative SHAP values indicate that an increase in the feature’s value decreases the model’s predictions, while positive SHAP values indicate that an increase in the feature’s value increases the model’s predictions [35]. 

## 3. Results and Discussion

In this section, descriptive statistics for the features and the results of the steps used to determine the most important features affecting *M. importuna* growth are presented.

### 3.1. Descriptive Statistics

The descriptive statistics for the 20 features and the growth of *M. importuna* are shown in Table 1, together with the minimum, maximum, mean, and standard deviation values.

According to Table 2, there are moderate and negative correlations between *M. importuna* growth and pH, lime (%), calcium, and high and negative correlations between *M. importuna* growth and phosphorus (ppm), and zinc (ppm). In other words, the increase in these substances in the soil causes a decrease in growth. In addition, there are moderate and positive correlations between *M. importuna* growth and magnesium (ppm), copper (ppm), and manganese (ppm). The increase in these substances in the soil causes an increase in growth.

### 3.2. Selection of Important Features

This section focuses on identifying the key features influencing prediction of *M. importuna*’s growth. A three-step procedure is followed to achieve the objective. In the first step, the data set consisting of 20 features and *M. importuna* growth is modeled by using eleven machine learning algorithms, namely AB, DT, XGB, ET, RF, GB, KNN, HGB, RR, LR, and LiR. In the second step, the model satisfying the best fit is identified by using three GoF measures. The procedure followed for this step is as follows. The data set is split into four subsets using the KFold [36] function. For this step, numerous random state values are tested, and the random state, training set, and test set that provided good performance in mean for all methods used in the study are determined. The random state value is 1667. There are 27 observations in the training set and 9 observations in the test set. The test set is used to choose the best fit algorithm based on GoF measures, whereas the training set is utilized to train machine learning algorithms. The goal is for the algorithms to generate predictions for the test set observations that are nearly accurate. This is because the algorithms’ performance on the test set will provide insight into how well they can predict the corresponding value of *M. importuna* growth when new features are introduced. In the third step, permutation importance and SHAP values are calculated based on the selected best-fit model to identify the features that contributed most to the predictions of *M. importuna* growth. No scaling, missing data imputation, or any other data preprocessing method was applied to the data set prior to the modeling process. Python 3.9 program, Scikit-learn (1.3.2), pandas (2.1.4), numpy (1.24.3), matplotlib, seaborn, openpyxl, random, SHAP, and XGBoost (2.0.3) are used for the analyses.

**Figure 4:**
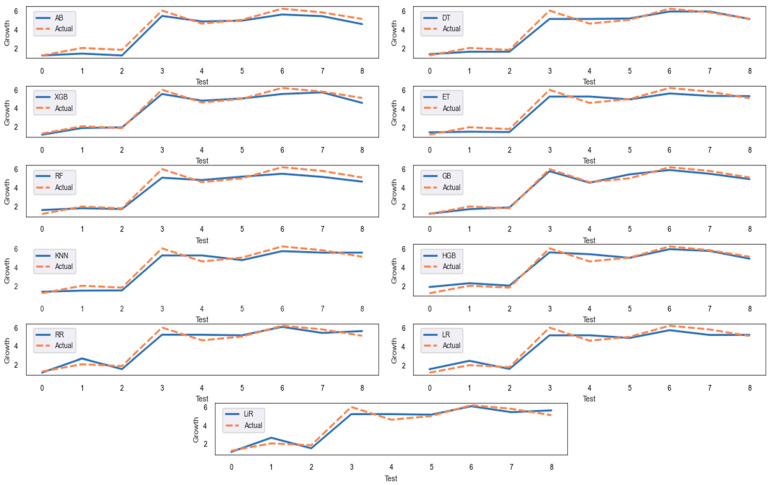
The actual and predicted values for the test set.

Figure 4 shows the actual and predicted values for the test set.

According to Figure 4, GB and XGB produce the prediction values closest to the actual values. Table 3 gives the GoF measures obtained from the eleven models for the test set.

**Figure 5:**
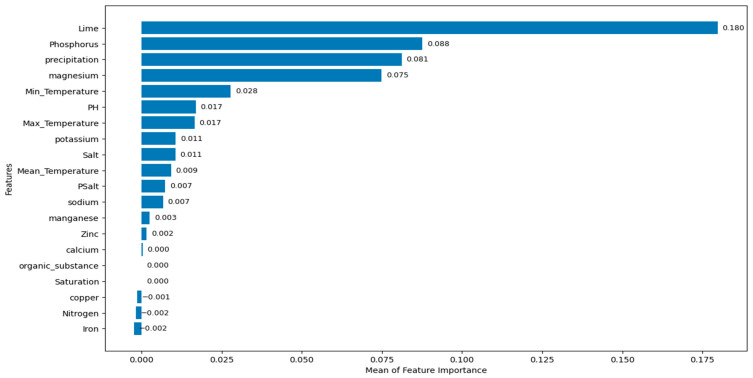
The mean of feature importance scores.

**Figure 6:**
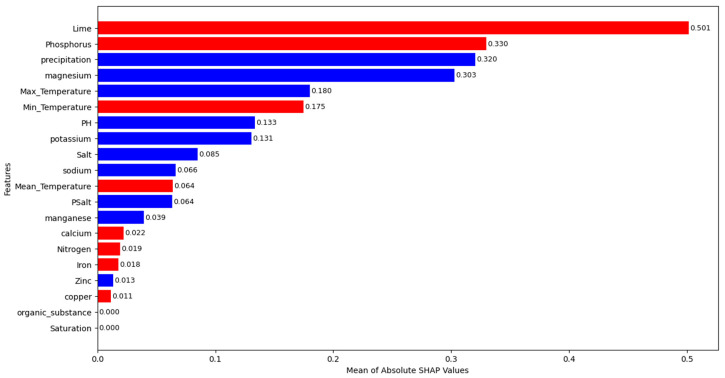
The mean of absolute SHAP values.

When looking at Table 3, it can be seen that GB provides the smallest MAE and MAPE values and the highest R^2^ value for the test set. XGB and DT also produce good prediction results. Based on GB, which offers the best fit for the test set, the most crucial features are identified by using permutation importance and SHAP. Mean of feature importance scores and SHAP values are given in Figure 5 and Figure 6, respectively. In Figure 6, SHAP values are presented as the mean of their absolute values to more clearly highlight the importance of the feature. The red bars in the figure indicate features with a negative SHAP mean, while the blue bars indicate features with a positive SHAP mean.

According to Figure 5, the mean of the feature importance scores of lime, phosphorus, precipitation, magnesium, minimum temperature, PH, and maximum temperature are 0.180, 0.088, 0.081, 0.075, 0.028, 0.017, and 0.017, respectively. The most important feature affecting prediction of *M. importuna* growth is the lime. Then, respectively, phosphorus, precipitation, and magnesium can be considered as effective features on prediction of *M. importuna* growth. The mean of feature importance of organic substance and saturation are found to be zero. This means that organic substance and saturation do not contribute significantly to the prediction of *M. importuna* growth. Feature importance means of copper, nitrogen, and iron are negative. These features increase the prediction error.

When looking at Figure 6, the highest mean of absolute SHAP value is provided by lime with 0.501. In addition, phosphorus (0.330), precipitation (0.320), and magnesium (0.303) are also found to significantly contribute to the predictions. Saturation (0.00) and organic substance (0.00) are not found to be significant features in terms of their effect on predictions based on mean absolute SHAP values. It is determined that copper (0.011), zinc (0.013), iron (0.018), nitrogen (0.019), calcium (0.022), and manganese (0.039) have small effect on the growth predictions. It is also possible to say from Figure 6 that increases in lime, phosphorus, and minimum temperature decrease the predictions of the growth, while increases in precipitation, magnesium, and maximum temperature increase the predictions of the growth. 

## 4. Conclusions

*M. importuna* is a mushroom species with high economic and nutritional value, but its cultivation faces challenges such as overharvesting, habitat loss, and ongoing uncertainty regarding the most suitable growing environment. This study aims to integrate machine learning with ecological and soil data to predict the growth of *M. importuna*. In other words, this study has quantitatively demonstrated the importance of environmental factors in *M. importuna* cultivation using machine learning. For this objective, we conducted a procedure with three steps. In the first step, the data set consisting of 20 features and *M. importuna* growth is modeled by using eleven machine learning algorithms, including AB, DT, XGB, ET, RF, GB, KNN, HGB, RR, LR, and LiR. The second step identifies the model satisfying the best fit by using three GoF measures. In the last step, the permutation importance and SHAP methods are executed to identify the features that have the most impact on predictions of growth. The following results are obtained:

- It is observed that the model that best explained the relationship between the 20 features measured from air and soil and the growth of the *M. importuna* is GB, according to the test set.
- Among the 20 evaluated features, lime (%) is found to be the most critical factor according to both permutation importance and SHAP methods.
- According to both feature importance methods, lime (%) is followed by phosphorus, precipitation, and magnesium in terms of its contribution to predictions. These findings are consistent with ecological observations and highlight the importance of both soil chemistry and climatic conditions in morel cultivation.
- There are no significant effects of the organic substance and the saturation on the predictions. Manganese, zinc, and calcium are features that have small effects on growth predictions. According to permutation importance scores, copper, nitrogen and iron increase the prediction error.
- According to SHAP values, an increase in lime, phosphorus, and minimum temperature decreases growth predictions for *M. importuna*, while an increase in precipitation, magnesium, maximum temperature, PH, and potassium increases them.
- The study has shown that an increase in maximum temperature increases growth predictions. This result may not be consistent with the literature [15]. However, the highest temperature observed during our measurement period was 22. Therefore, it will not be correct to say that growth predictions will increase at high temperatures.
- It has been determined that increases in minimum and mean temperature values will decrease the growth predictions. Again, the maximum values of minimum and mean temperatures obtained during the measurement period were measured as 16.2 and 18.5, respectively. This part of the study is consistent with the literature [15].
- Correlation analysis (Table 2) found a moderate negative correlation (−0.57) between pH and calcium with the growth, a high negative correlation (−0.85) between zinc and the growth, and a moderate positive correlation (0.57) between copper and manganese with the growth. These correlations contradict the SHAP results. However, the correlation analysis assumed the independence of the features and investigated their linear correlation.
- Practically, the results provide quantitative guidance for optimizing cultivation strategies. For example, monitoring lime, phosphorus, magnesium concentration, and temperature regimes could significantly enhance growth outcomes. Additionally, these insights may inform conservation efforts by identifying habitats most favorable for sustainable harvesting. The integration of machine learning into fungal cultivation studies could open new opportunities for precision agriculture and biotechnology.

## Figures and Tables

**Table 1:** Descriptive statistics of the features used in the study.

		Minimum	Maximum	Mean	Std. Deviation
X1	Saturation (%)	34.80	51.00	41.33	7.07
X2	Saltness (dS/m)	0.28	0.38	0.32	0.04
X3	Salt (%)	0.01	0.01	0.01	0.00
X4	pH	7.19	8.13	7.77	0.42
X5	Lime (%)	1.43	22.35	12.07	8.67
X6	Organic Substance (%)	1.13	3.58	2.34	1.01
X7	Nitrogen (%)	0.06	0.18	0.12	0.05
X8	Phosphorus (ppm)	9.00	18.00	12.67	3.91
X9	Potassium (ppm)	96.42	115.01	106.36	7.75
X10	Calcium (ppm)	4750.99	8863.06	7473.53	1952.57
X11	Magnesium (ppm)	85.46	163.97	120.92	32.96
X12	Sodium (ppm)	41.84	49.73	46.81	3.58
X13	Iron (ppm)	64.46	116.61	90.93	21.60
X14	Copper (ppm)	0.92	1.03	0.97	0.05
X15	Manganese (ppm)	85.90	121.60	104.16	14.79
X16	Zinc (ppm)	0.37	0.77	0.59	0.17
X17	Mean Temperature	12.70	18.50	15.00	1.77
X18	Max Temperature	17.40	22.00	19.28	1.19
X19	Min Temperature	5.80	16.20	11.15	3.13
X20	Precipitation	0.00	12.10	2.38	3.84
Y	*M. Importuna* Growth (cm)	1.20	6.20	3.91	1.78

The correlations between *M. importuna* growth and the features are given in Table 2.

**Table 2:** The correlations between the features of *M. importuna* in habitat soil features and growth.

Feature	Growth	Feature	Growth
Saturation	−0.28	Magnesium (ppm)	0.57
Salt	−0.28	Sodium (ppm)	0.28
Salt (%)	−0.28	Iron (ppm)	0.28
pH	−0.57	Copper (ppm)	0.57
Lime (%)	−0.57	Manganese (ppm)	0.57
Organic Substance (%)	−0.28	Zinc (ppm)	−0.85
Nitrogen (%)	−0.28	Mean Temperature	0.01
Phosphorus (ppm)	−0.85	Max Temperature	−0.07
Potassium (ppm)	0.28	Min Temperature	0.03
Calcium (ppm)	−0.57	Precipitation	0.26

**Table 3:** GoF measures for test set.

	MAE	R^2^	MAPE
AB	0.4085	0.9365	0.1196
DT	0.2970	0.9550	0.0861
XGB	0.2647	0.9661	0.0664
ET	0.4172	0.9359	0.1242
RF	0.4210	0.9275	0.1148
GB	0.2100	0.9827	0.0539
KNN	0.4278	0.9369	0.1213
HGB	0.3287	0.9523	0.1283
RR	0.4041	0.9373	0.1196
LR	0.4168	0.9349	0.1271
LiR	0.4064	0.9368	0.1215

## Data Availability

Data is provided within the manuscript.
